# Dr. Julian Taintor Williams

**Published:** 1905-05

**Authors:** 


### OBITUARY.

DR. JULIAN TAINTOR WILLIAMS, of Dunkirk, died at his home April 10, 1905, aged 75 years. Dr. Williams was born at Dunkirk, November 15, 1829. He graduated at Fredonia academy and afterward entered Buffalo University as a student of medicine. He graduated at Vermont medical college, Castleton, in 1851, and at once began practice at Dunkirk, in which he continued for thirty years. He was twice a member of the state legislature, first in 1865, and again in 1885. He retired from practice some years ago and at the time of his death was editor of the *Dunkirk Evening Observer.*

